# Identification of a Rare Variant of c.1777G>A (p.G593S) in the *COL1A1* Gene as the Etiology of Recurrent Osteogenesis Imperfecta by Whole-Exome Sequencing

**DOI:** 10.3389/fped.2022.816090

**Published:** 2022-04-08

**Authors:** Jianlong Zhuang, Chunnuan Chen, Yu'e Chen, Qi Luo, Yuanbai Wang, Yuying Jiang, Shuhong Zeng, Yingjun Xie, Dongmei Chen

**Affiliations:** ^1^Prenatal Diagnosis Center, Quanzhou Women's and Children's Hospital, Quanzhou, China; ^2^Department of Neurology, The Second Affiliated Hospital of Fujian Medical University, Quanzhou, China; ^3^Department of Ultrasound, Quanzhou Women's and Children's Hospital, Quanzhou, China; ^4^Department of Public Health for Women and Children, Quanzhou Women's and Children's Hospital, Quanzhou, China; ^5^Key Laboratory for Major Obstetric Diseases of Guangdong Province, Department of Obstetrics and Gynecology, The Third Affiliated Hospital of Guangzhou Medical University, Guangzhou, China; ^6^Key Laboratory of Reproduction and Genetics of Guangdong Higher Education Institutes, The Third Affiliated Hospital of Guangzhou Medical University, Guangzhou, China; ^7^Department of Neonatal Intensive Care Unit, Quanzhou Women's and Children's Hospital, Quanzhou, China

**Keywords:** osteogenesis imperfecta, type I collagen, *COL1A1*, whole-exome sequencing, chromosomal microarray analysis

## Abstract

**Background:**

Osteogenesis imperfecta (OI) is a rare heterogeneous disorder typically featured by fragile bones and susceptibility to fracture. The aim of the present study was to explore the genetic etiology of familial recurrent OI and the genotype–phenotype correlation.

**Methods:**

Karyotyping, chromosomal microarray analysis, and whole-exome sequencing (WES) were performed to determine the genetic etiology of OI in the enrolled family. Western blotting analysis was carried out using the fetal skin tissue for type I collagen production analysis.

**Results:**

At the first pregnancy, a c.1777G>A mutation in the *COL1A1* gene was detected in the fetus who exhibited skeletal dysplasia. In this second pregnancy, severe fetal skeletal dysplasia was also presented without significant chromosomal abnormality detected by karyotype and chromosomal microarray analysis in the fetus. Further WES results demonstrated a *de novo* missense mutation of c.1777G>A (p.G593S) in the fetus, which was classified as a pathogenic variant according to the ACMG guidelines. The recurrent mutation in the two fetuses hinted at the possible existence of gonadal mosaicism in the parents, while no mutation in the *COL1A1* gene was identified in the DNA from the father's sperm. In addition, Western blot results demonstrated no reduced type I procollagen production in the affected fetus compared with the age-matched controls.

**Conclusions:**

To the best of our knowledge, this is the first study that identified a rare variant of c.1777G>A in the *COL1A1* gene that led to recurrent OI in the Chinese population. Additionally, we believe that this rare variant of c.1777G>A in the *COL1A1* gene will lead to OI type II. The results of the present study further verify the application value of WES in identifying fetuses with ultrasound anomalies.

## Introduction

Osteogenesis imperfecta (OI), also known as brittle bone disease, is a disorder of connective tissue typically featured by fragile bones and susceptibility to fracture, mostly (~ 90%) caused by autosomal dominant pathogenic variants in the *COL1A1* and *COL1A2* genes, which encode type I collagen (1, 2). In 1979, Sillence et al. (3) proposed to classify OI into four categories based on the severity of the clinical manifestations, which has become the standard classification of OI. Type I shows mild clinical features with blue sclerae, near-normal stature, and without obvious dentinogenesis imperfecta; type II is the most severe type that leads to perinatal lethality with blue sclerae; type III results in a severe type, typically exhibiting a progressive deforming variety without blue sclerae; and type IV is a moderate type with normal sclerae, demonstrating a more severe phenotype than mild OI type I (2). The overall incidence of OI is 0.5~1 per 10,000 individuals (4, 5). It is reported that the birth prevalence ranges from 3/100,000 to 7/100,000 in Europe and the United States (6, 7); 0.5 per 10,000 in Finland, with most of them harboring milder OI type I and type IV (8); and 1 per 100,000 people in Vietnam, who present with more severe phenotypes (9). However, there is still a lack of epidemiologic information on OI in the Chinese population at present.

Type I collagen is an important component of most connective tissues and bones of the human body, is the major protein component of the extracellular matrix in bones, skin, and tendons, and is mainly secreted by osteoblasts, dermal fibroblasts, and tenocytes (2). The type I collagen triple helix consists of two α1 chains and one α2 chain, which plays an important role in the stability of the whole collagen molecular structure. Moreover, the triple-helix region of each peptide chain contains 338 consecutive repeats of Gly-Xaa-Yaa, with Gly being the essential amino acid in this sequence and the one able to maintain the normal spatial structure of the triple helix (10). In general, mutations in the *COL1A1* gene elicited a more severe clinical phenotype than *COL1A2* gene mutations (11, 12), which may be ascribed to the type I collagen protein molecular formula that implies the presence of a mutant chain in 75% and 50% of collagen triple helices from defects in α1 and α2, respectively (2).

An increased number of patients with OI have been identified using whole-exome sequencing (WES) technology. In this study, we first detected a rare variant of c.1777G>A in the *COL1A1* gene that led to recurrent OI in the Chinese population using WES technology, which may provide valuable data for genetic consultation and highlight the application value of WES in the genetic screening of fetuses with ultrasound anomalies.

## Materials and Methods

### Subjects

**Figure 1:**
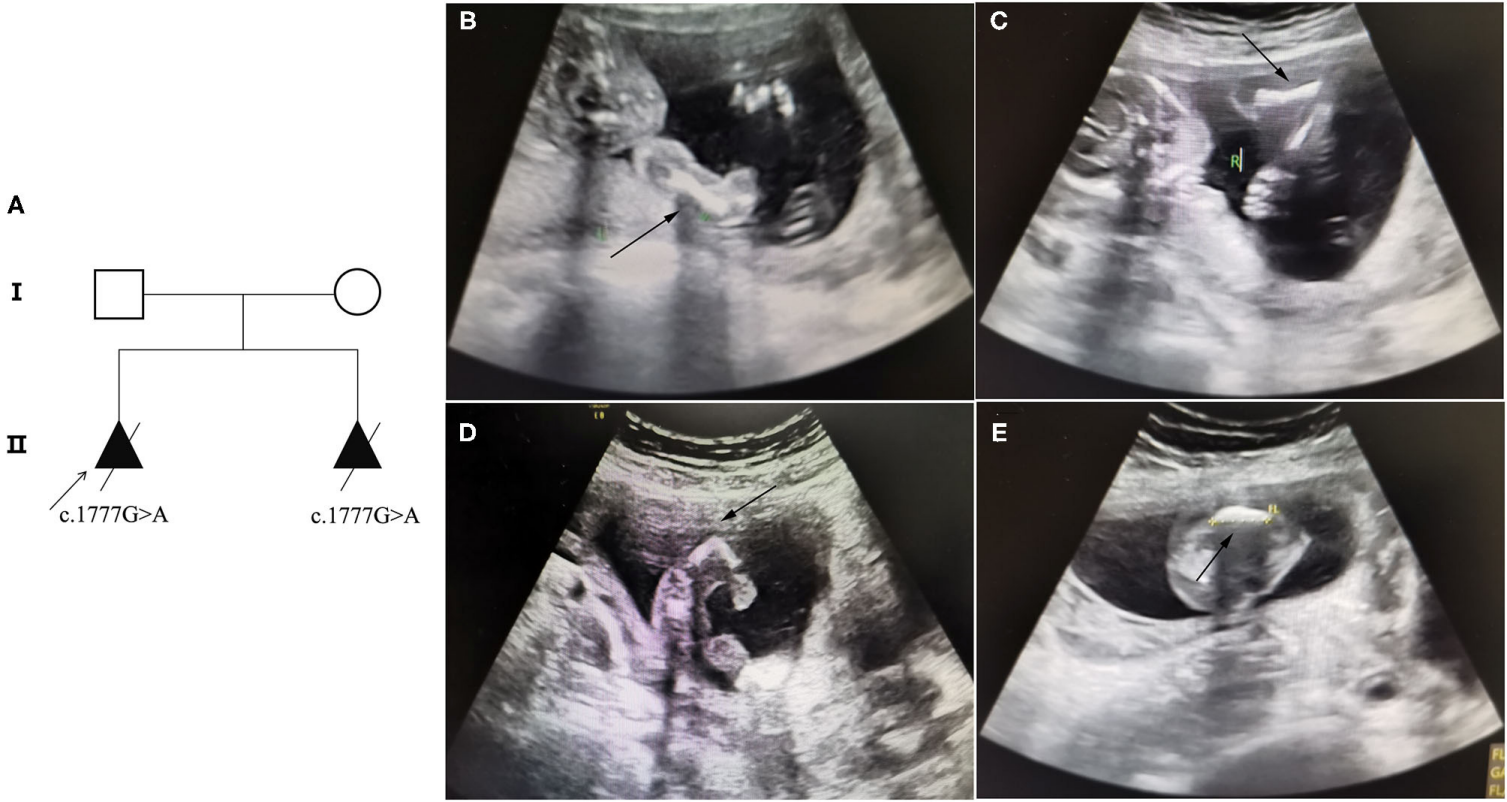
Pedigree information and ultrasound anomalies detected in the fetus. **(A)** Pedigree information in this family. **(B,C)** Ultrasound results showing that the bilateral humerus of the fetus was slightly curved. **(D)** The ultrasound results revealing that the left femur of the fetus was shorter and bent into an angle. **(E)** The ultrasound results suggested that the right femur of the fetus was shorter and slightly curved. The arrows indicate the anomalies.

A 30-year-old pregnant woman with an adverse pregnancy history and prenatal ultrasound anomalies came to Quanzhou Women's and Children's Hospital for genetic consultation. Two pregnancies in this family displayed fetal OI (Figure 1A). The couple denied any consanguinity. Written informed consent was obtained from the family, and the study protocol was approved by the Ethics Committee of Quanzhou Women's and Children's Hospital (2020 No.31).

In this study, recurrent OI was defined as identification of OI in one affected pregnancy/infant, and after two or more affected pregnancies/infants with OI or independently two or more pregnancies/infants harboring the same OI-associated gene variant.

### Karyotype Analysis

Approximately, 10 ml of amniotic fluid and 2 ml of peripheral blood were collected from the fetus and parents, respectively. Chromosomes were prepared according to the karyotype operation procedure in the prenatal diagnosis department of the said hospital using the automatic chromosome harvesting system Sinochrome Chromprep II (Lichen Biotechnology Co., Ltd., Shanghai, China) (13). The karyotype nomenclature and diagnosis were conducted following ISCN 2020.

### Chromosomal Microarray Analysis

Approximately, 3~5 ml of peripheral blood was collected from the parents. The subjects' DNA was extracted by SNP array and WES analysis using a QIAamp DNA Blood Kit (Qiagen, Hilden, Germany) following the instructions from the kit's handbook (www.qiagen.com). Analysis of the SNP array was carried out using the Affymetrix CytoScan 750K Chip Kit following the standard procedure reported previously (14). Copy number variants were interpreted with reference to DGV, OMIM, DECIPHER, and PubMed and other databases.

### WES and Data Analysis

DNA quantification was performed using the Qubit dsDNA HS Assay (Invitrogen, Carlsbad, CA, USA). Covaris LE220 (Covaris, Woburn, MA, USA) was used to shear the DNA to an approximate mean fragment length of 150–200 bp. WES was performed based on the Illumina HiSeq 2500 platform (Illumina, San Diego, CA, USA).

Data analysis included variant calling, annotation, and variant screening. Pretreatment for variant calling comprised the following: (1) the raw fastq files were trimmed using Fastp (v.0.12.0, -N 15-L 30), and the low-quality reads were filtered out from all the sequencing dates using a quality score of 20. (2) The filtered sequencing data were mapped to the human reference genome (hg19) using BWA (v.0.7.17-r1188) with the MEM alignment method. (3) The SAM file was converted into a compressed binary BAM file by Picard-SortsAM. (4) The sample was given an ID for matched reads by Picard-Add or Replace Read Groups. (5) The same sequences created by PCR duplication and optical duplication by Picard-Mark Duplicates were marked. (6) GATK-Baserecalibrator and GATK-ApplyBQSR were used for site quality correction according to the known SNP and InDel sites of 1000 Genomes Projects. And (7) GATK-Haplotypecaller was used for variant identification and formation of a VCF file. Variants were annotated on public databases by ANNOVAR based on gene, region, and filter in the following steps. (1) Gene-based annotation identified whether SNPs caused protein-coding changes and revealed the amino acids that are affected. (2) Region-based annotation depicted variants in specific genomic regions. And (3) Filter-based annotation identified variants that were documented in the population frequency databases, including 1000 Genomes Project, Exome Aggregation Consortium, and Genome Aggregation Database (gnomAD). All whole-exome variants were subjected to biological effect analysis, using the programs including SIFT, MutationTaster, PolyPhen-2, Human Splicing Finder, and MaxEntScan. Other annotation databases including OMIM, ClinVar, and InterVar were used to determine mutation harmfulness and pathogenicity. The detected variants were classified as pathogenic, likely pathogenic, VOUS, likely benign, or benign based on the ACMG guidelines (15). Candidate variants were selected after combining the clinical information of the proband and the above-annotated information, placing emphasis on *de novo* variants, compound heterozygotes, homozygotes, and hemizygotes.

### Western Blotting Analysis

The fetal abortion skin tissues in the proband and age-matched skin tissues in the control group were obtained. All the tissues were extracted using the T-PER Tissue Protein Extraction Reagent (78510, Thermo Pierce, Waltham, MA, USA) and qualified by BCA assay (P0010s, Beyotime, Shanghai, China). Subsequently, 60 μg of total protein was loaded per lane on 8%~12% SDS-PAGE gels and transferred to polyvinylidene fluoride membranes to perform Western blotting analysis (PVDF, Bio-Rad, Hercules, CA, USA). Type I collagen that was extracted from the intracellular collagen was detected using the collagen I antibody (Abcam, Cambridge, MA, USA, ab138492). GAPDH was used as an internal reference (Abcam, ab181602).

## Results

### Case Information

Recruited in this study was a 30-year-old pregnant woman, gravida 2, para 0, from Quanzhou city, Fujian province, China, with an adverse pregnancy history and prenatal ultrasound anomalies. During her first pregnancy, a c.1777G>A mutation in the *COL1A1* gene was detected in the fetus who exhibited skeletal dysplasia, thus the family chose to terminate her pregnancy. In this second pregnancy, skeletal dysplasia was also depicted, presenting as short and curved femurs and humerus in the fetus screening by ultrasound examination at the gestational age of 19 weeks (Figures 1B–E). After prenatal genetic consultation and signing of informed consent, amniocentesis was conducted at the gestational age of 20 weeks. A review of the *COL1A1* mutations in exon 26 is displayed in Table 1.

**Table 1:** Mutations of the *COL1A1* gene in exon 26 were reviewed in the ClinVar database.

**Mutations**	**Protein**	**Exon**	**Clinical phenotype**	**Mutation types**	**Clinical significance**
c.1821+1G>T	/	26	OI type I	Splicing mutation	Pathogenic
c.1821+1G>C	/	26	OI type I	Splicing mutation	Pathogenic
c.1821+1G>A	/	26	OI type I/III	Splicing mutation	Pathogenic
c.1821del	p.Gly608fs	26	OI type I	Frameshift mutation	Pathogenic
c.1812del	p.Gly605fs	26	OI	Frameshift mutation	Likely pathogenic
c.1804G>T	p.Gly602Ter	26	OI type I	Nonsense mutation	Pathogenic
c.1797del	p.Val600fs	26	OI type I	Frameshift mutation	Pathogenic
c.1792C>T	p.Arg598Ter	26	OI type I	Nonsense mutation	Pathogenic
c.1777G>T	p.Gly593Cys	26	OI type III/IV	Missense mutation	Pathogenic
c.1777G>A	p.Gly593Ser	26	OI type II/III	Missense mutation	Pathogenic
c.1772_1773del	p.Glu591fs	26	OI type I	Frameshift mutation	Pathogenic
c.1768-1G>A	/	26	OI type I	Splicing mutation	Pathogenic

### Karyotyping and Chromosomal Microarray Analysis in the Family

Karyotype analysis did not elicit a significant chromosomal abnormality in either the fetus or the parents. Moreover, chromosomal microarray analysis showed no copy number variants in the family.

### WES Detection in the Family

**Figure 2:**
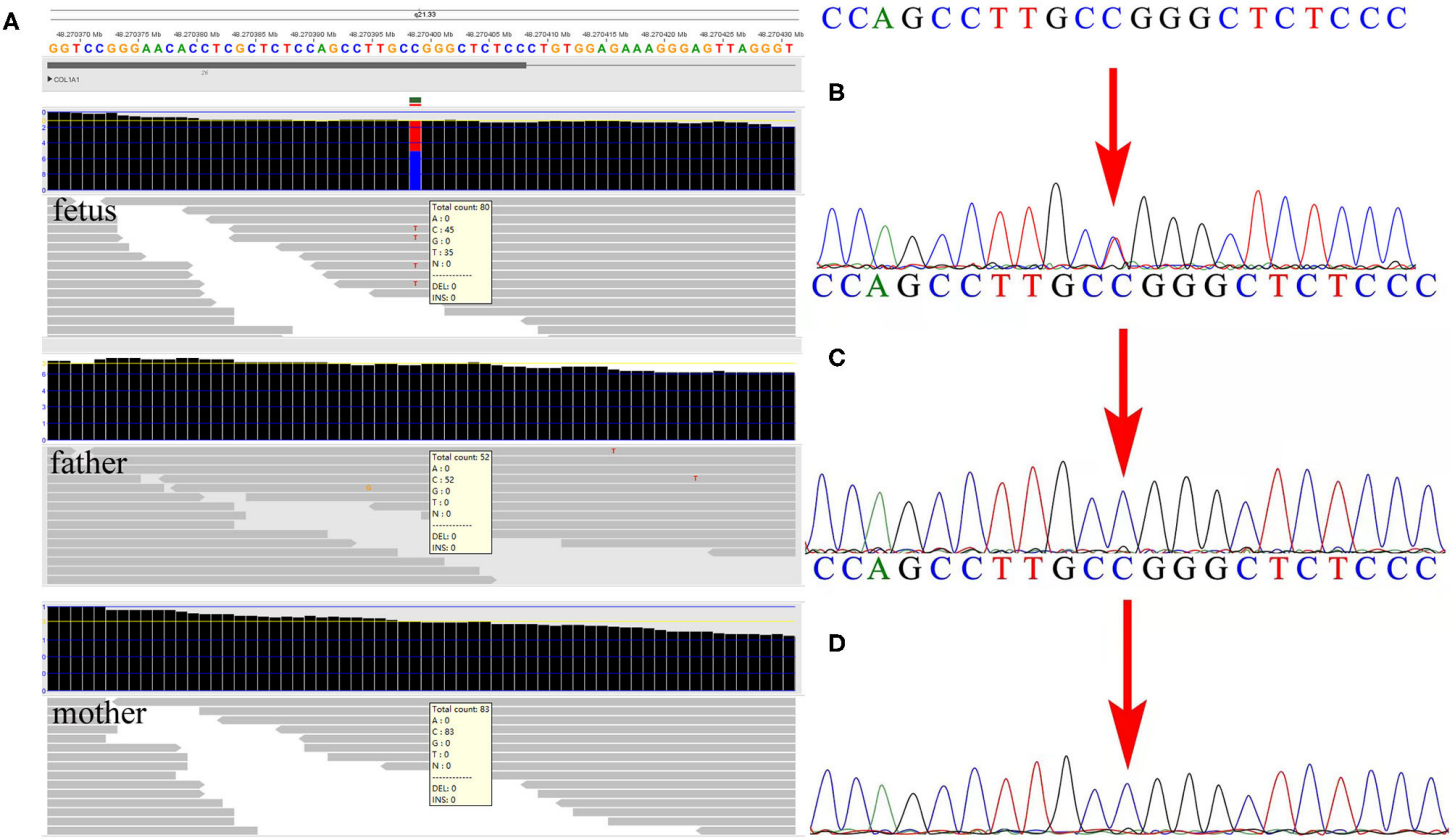
Detection of the variant by whole-exome sequencing (WES) and verification by Sanger sequencing in the family. **(A)** The WES detection results demonstrated a *de novo* variant of c.1777G>A in exon 26 of the *COL1A1* gene in the fetus. **(B)** Sanger sequencing verified the variant detected by WES in the fetus. **(C,D)** Neither parent carried the c.1777G>A mutation in the *COL1A1* gene by Sanger sequencing.

Subsequent WES technology results from the family demonstrated a *de novo* missense mutation of c.1777G>A (p.G593S) in the fetus but not in the parents (Figure 2A), which were further verified by Sanger sequencing (Figures 2B–D). Furthermore, none of the other variants were observed in *COL1A1/A2* genes, or other OI-associated genes. No frequency of p.G593S was reported in the common population databases including ExAC, 1000 Genome, and gnomAD. Several mutation hazard prediction software programs suggest that the mutation would possibly affect the protein structure or function (MetaSVM_score: 0.961; GERP++_rs: 4.35). The variant was classified as pathogenic according to the ACMG guidelines (PS2_very strong, PM5, PM2_supporting, PP3). The variant of c.1777G>A in the fetus was consistent with that in the first fetus of this family, suggesting possible gonadal mosaicism in the parents. Further investigation revealed that none of the mutations in the *COL1A1* gene were observed in paternal sperm DNA, which was highly suggestive of maternal gonadal mosaicism, but unfortunately, maternal gonadal tissue was not available in this study.

### Type I Collagen Product Expression Analysis

**Figure 3:**
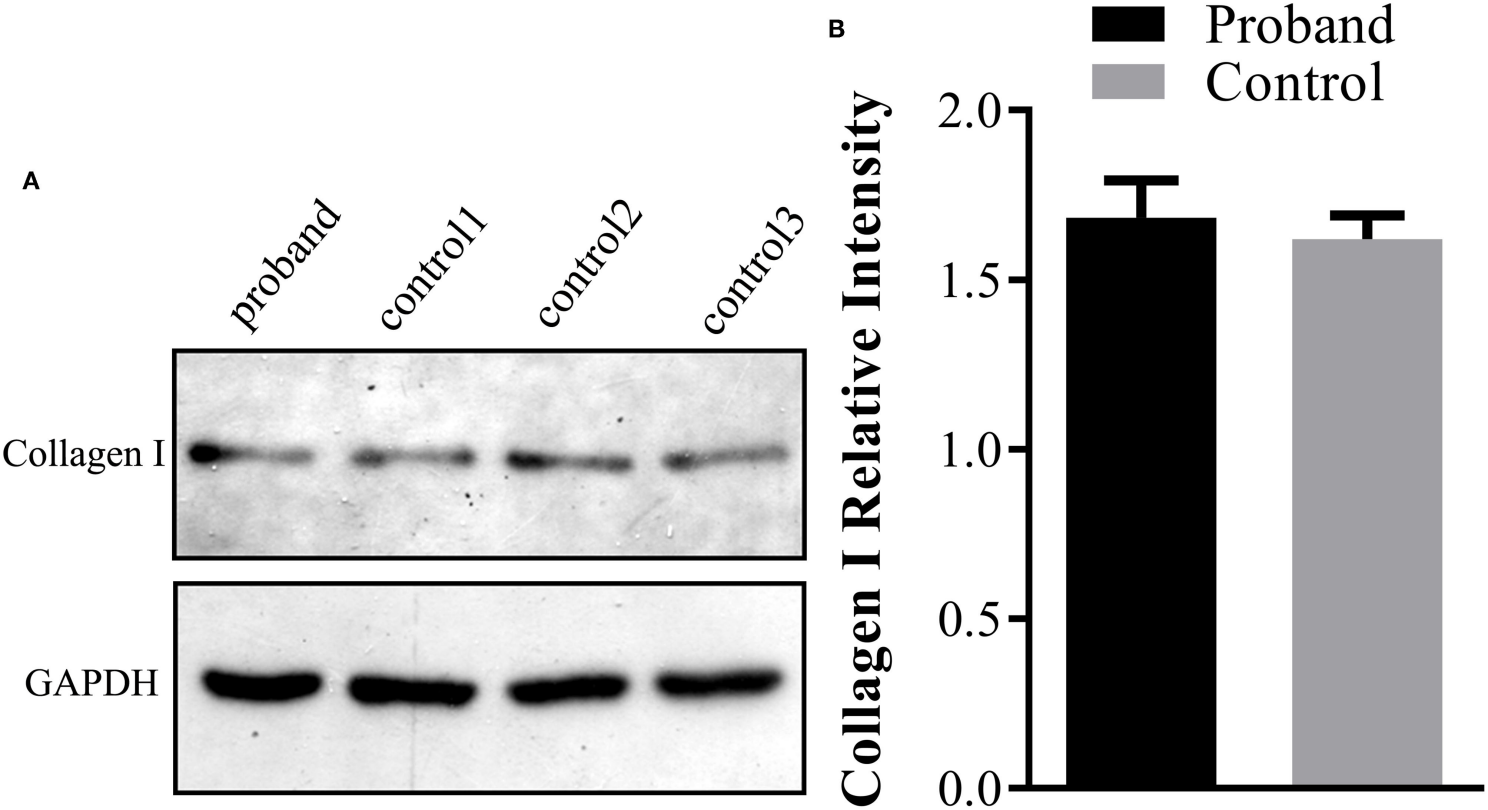
Type I collagen production assay using Western blot analysis in the fetus. **(A)** The protein was extracted from the fetal abortion skin tissue for Western blot analysis, and three age-matched skin tissues without the *COL1A1* mutation were used as the controls. **(B)** No significant reduction of the intracellular type I collagen product was observed in the proband compared with the control group.

Further Western blotting analysis was carried out using the fetal skin tissue for intracellular type I collagen production analysis, using three cases of age-matched skin tissues as controls. The results demonstrated that no significant reduction of the type I collagen product was observed in the affected fetus compared with the controls (Figure 3).

## Discussion

Type I collagen is the main component of the bone extracellular matrix (16). Its quantity and quality are important determinants of bone strength, which are affected by *COL1A1* and *COL1A2* gene mutations. Among them, *COL1A1* mutation is the most dominant with more than 1,000 different mutations reported in the databases (17, 18).

In the present study, we first identified a *de novo* variant of c.1777G>A in the *COL1A1* gene of the proband that exhibited an extremely severe clinical phenotype, which may lead to perinatal lethality (OI type II). A previous study (19) reported that OI type II was commonly caused by *de novo* mutations, which is consistent with our results. Additionally, it was found that recurrent OI type II in the affected offspring usually results from parental gonadal mosaicism (20, 21). In this study, we identified a *de novo* variant of c.1777G>A in the *COL1A1* gene that resulted in recurrent OI, suggesting that parental gonadal mosaicism may exist in this family. Therefore, we performed DNA sequencing by using genomics DNA extracted from paternal sperm cells. However, the results delineated no mutation in the *COL1A1* gene. Although maternal gonadal mosaicism was highly suggestive, unfortunately no maternal gonadal tissue was available in this study.

To the best of our knowledge, only two studies have reported this rare c.1777G>A mutation (p.G593S) (21, 22). The first study reporting this rare mutation was published in 1992 and hypothesized that the mutation may cause OI type III/IV, but the child died at 19 months (22). Another study reported the same mutation again in 1993 (21). Interestingly, they observed a severe recurrent OI type II/III resulting from paternal gonad mosaicism. Finally, the present study is the third publication reporting the rare variant of c.1777G>A, in which familiar recurrent OI was observed, which we believe may lead to OI type II, because severe skeletal dysplasia was detected in the fetus by ultrasound. Using Western blotting analysis, we extracted proteins from the fetal skin tissue to explore the expression of the type I collagen product in the affected fetus, but no significant reduction in the expression of the type I collagen product was seen compared with the age-matched control, which is consistent with the previous study (22). Additionally, an overview of our study and previous literature reports showed that the c.1777G>A mutation in recurrent OI resulted from parental gonadal mosaicism, which is also horizontal proof that c.1777G>A mutation will lead to severe OI type II.

There are two general classes of mutations in *COL1A1/A2* that lead to OI (1). The mutations that quantitatively reduce type I collagen will result in milder OI type I; such mutations include nonsense mutations, frameshift mutations, and splicing. Other mechanisms affecting structural collagen defects cause more severe OI through a substitution for glycine in the Gly-Xaa-Yaa repeat in the triple helix. The study first identified the p.G593S mutations (22), showing a slow migration of type I collagen α1(I) and α2(I) chains, and reduced thermal stability of type I collagen compared with the controls. Additionally, fibroblast type I collagen production was not reduced, and the type I collagen secretion was also not noticeably impaired in comparison with the controls. However, the second study by Mottes et al. (21) reported the same mutation, indicating a lower secretion of type I procollagen than controls. Here, we identified the same mutation in a Chinese family; further Western blot analysis indicated that the type I collagen protein was not notably reduced compared with the controls, while the secretion of type I collagen was not available in this study. Additionally, as delineated in Table 1, most variants in exon 26 of the *COL1A1* gene exhibited OI type I, with premature mutations, whereas the mutation associated with glycine of the Gly-Xaa-Yaa repeat in the triple helix in our study elicited a more severe phenotype, which was also consistent with the findings published by previous studies in the literature (21).

A previous study reported (23) a 27% recurrence risk in couples after two or more affected fetuses with perinatal lethal OI in families with parental mosaicism. Although our study did not further verify the existence of maternal gonadal mosaicism, maternal gonadal mosaicism is highly suggestive in this family. According to the recurrence risk of the mutation and after careful genetic consultation, we propose that prenatal or preimplantation genetic diagnosis should be provided in the next pregnancy.

In conclusion, this study is the first to identify a rare variant of c.1777G>A in exon 26 of the *COL1A1* gene that causes recurrent OI in the Chinese population. Furthermore, our study further verified that the rare variant of c.1777G>A in the *COL1A1* gene would lead to OI type II and highly suggests that recurrent OI may ascribe to maternal gonadal mosaicism in this family. Moreover, our study provides valuable data for genetic consultation and confirms the application value of WES in the genetic screening of fetuses with ultrasound anomalies.

## Data Availability Statement

The raw data supporting the conclusions of this article will be made available by the authors, without undue reservation.

## Ethics Statement

The studies involving human participants were reviewed and approved by Ethics Committee approval was obtained from the Institutional Ethics Committee of Quanzhou Women's and Children's Hospital to the commencement of the study (2020No.31). The patients/participants provided their written informed consent to participate in this study. Ethical review and approval was not required for the animal study because Ethics Committee approval was obtained from the Institutional Ethics Committee of Quanzhou Women's and Children's Hospital to the commencement of the study (2020No.31).

## Author Contributions

JZ designed the study. JZ and CC wrote the article. YC, QL, and YJ recruited the participants and performed the clinical consultation. YW and SZ performed the karyotype analysis and analyzed the data. DC and YX revised and polished the paper. All authors approved the final article.

## Funding

This research was supported by the Fujian Provincial Health Commission Youth Science and Technology Project (2020QNB045) and Quanzhou City Science and Technology Project (2020C026R).

## Conflict of Interest

The authors declare that the research was conducted in the absence of any commercial or financial relationships that could be construed as a potential conflict of interest.

## Publisher's Note

All claims expressed in this article are solely those of the authors and do not necessarily represent those of their affiliated organizations, or those of the publisher, the editors and the reviewers. Any product that may be evaluated in this article, or claim that may be made by its manufacturer, is not guaranteed or endorsed by the publisher.
